# Cryptococcal Pneumonia in Multiple Myeloma: A Case Report

**DOI:** 10.7759/cureus.75557

**Published:** 2024-12-11

**Authors:** Dustin Epstein, Joanna Herrera, Tiffany Nong, Stephanie Ocejo, Jeremy I Purow, Sandra Sepulveda, Jannelle Vicens, Marco Ruiz

**Affiliations:** 1 Oncology, Florida International University, Herbert Wertheim College of Medicine, Miami, USA; 2 Oncology, Florida State University College of Medicine, Tallahassee, USA; 3 Infectious Disease, Florida International University, Herbert Wertheim College of Medicine, Miami, USA; 4 Malignant Hematology/Bone Marrow Transplant/HIV Oncology, Miami Cancer Institute, Miami, USA; 5 Hematology Oncology, Miami Cancer Institute, Miami, USA; 6 Translational Medicine, Florida International University, Herbert Wertheim College of Medicine, Miami, USA

**Keywords:** cryptococcal pneumonia, immunocompromised patient, lung nodules, multiple myeloma, renal impairment

## Abstract

Patients with multiple myeloma (MM) often experience infections due to aberrant immunoglobulin production by malignant plasma cells and immunosuppressive therapeutic interventions that are used to treat the condition. A rare but serious infection that may occur in these patients is Cryptococcus, an encapsulated fungus that typically infects immunocompromised individuals. Cryptococcus infections often present as pneumonia but can disseminate to the central nervous system, potentially causing meningitis. Therefore, Cryptococcus poses a significant threat and warrants prompt recognition and treatment. We present a case of a 75-year-old male being assessed for relapsed MM with extensive nodular mass-like opacities in the lungs on PET CT. A few months later, the patient presented to the emergency department with complaints of sudden onset abdominal pain, chills, and fatigue. A CT scan confirmed the presence of bilateral lung consolidations. It was subsequently established that the patient was likely experiencing a relapse of MM prompting adjustments to their treatment plan. Laboratory testing indicated that the patient's light chains and renal function improved with the altered therapy, but the patient had minimal improvement for the bilateral lung consolidations. One month later, the patient presented to the infusion visit with a cough and sore throat. Blood titers were positive for cryptococcal antigen, indicating a potential cause for the pulmonary infiltration. The team considered bronchoscopy or lung nodule biopsy to confirm the diagnosis; however, given the patient's frailty, they opted to monitor the clinical response and repeat a PET-CT scan in seven days. Additionally, cerebrospinal fluid analysis following a lumbar puncture was negative. The patient was started on oral fluconazole, leading to symptomatic improvement and resolution of pulmonary nodules on chest CT.

## Introduction

Patients with multiple myeloma (MM) often have an impaired ability to respond to various forms of infections due to a significant tumor burden and antineoplastic therapy that impairs the immune system [1,2]. Impaired immune functioning in these patients is due to hypogammaglobulinemia, neutropenia, and lymphopenia. In addition to cellular and immunoglobulin deficiencies, multiple myeloma may lead to mucosal defects and respiratory dysfunction, further contributing to the susceptibility to infection in these patients. MM is also treated with immunosuppressive agents, which also predisposes these patients to various infections. In a retrospective analysis of 3,107 patients with MM, infection was the cause of death in 45% of the patients who died following the initiation of treatment [3].

Cryptococcus spp. is an encapsulated opportunistic fungal pathogen primarily associated with T-cell immunodeficiency, often linked to various conditions or medications that compromise immune function [1,4-6]. The infection is often treated empirically with broad-spectrum antifungals despite varying degrees of success with these treatments [5]. These treatments also have a high degree of toxicity. Regardless, it is essential to promptly and accurately identify patients with Cryptococcus to initiate treatment early in the clinical course.

Cryptococcus infection is rare in immunocompetent patients, and patients with disseminated Cryptococcus infection are almost exclusively immunodeficient [6]. Given the immunodeficiencies inherent in MM, it is plausible that such patients may be susceptible to varying degrees of Cryptococcal infections. However, Cryptococcal infection remains relatively uncommon in MM, as there are only a few reported cases with varying degrees of dissemination in the literature [7]. Among the few patients with both multiple myeloma and cryptococcal infection, immunosuppression - primarily evidenced by leukopenia, but also by hypogammaglobulinemia - would be expected. We report a case of Cryptococcal pneumonia in a patient with MM.

## Case presentation

The patient is a 75-year-old male with relapsed/refractory RISS Stage II, high-risk IgG lambda multiple myeloma, who was originally diagnosed in July 2020. Upon diagnosis, the patient presented with an abnormal cytogenetic profile, including a male karyotype with the loss of eight “Y” chromosomes and gains in the 1q and 15q chromosomal regions.

The patient underwent several lines of therapy, including Bortezomib, Lenalidomide, and Dexamethasone, for six cycles. The therapy was then changed to Carfilzomib, Pomalidomide, and Dexamethasone for five cycles prior to autologous hematopoietic stem cell transplantation. The patient had a transplant in August of 2021. In December of 2021, the patient was started on maintenance therapy with Lenalidomide. Following transplantation, the patient had a relatively uncomplicated clinical course and recovery besides the development and progression of Alzheimer’s dementia. Brain imaging was mostly unremarkable.

**Figure 1:**
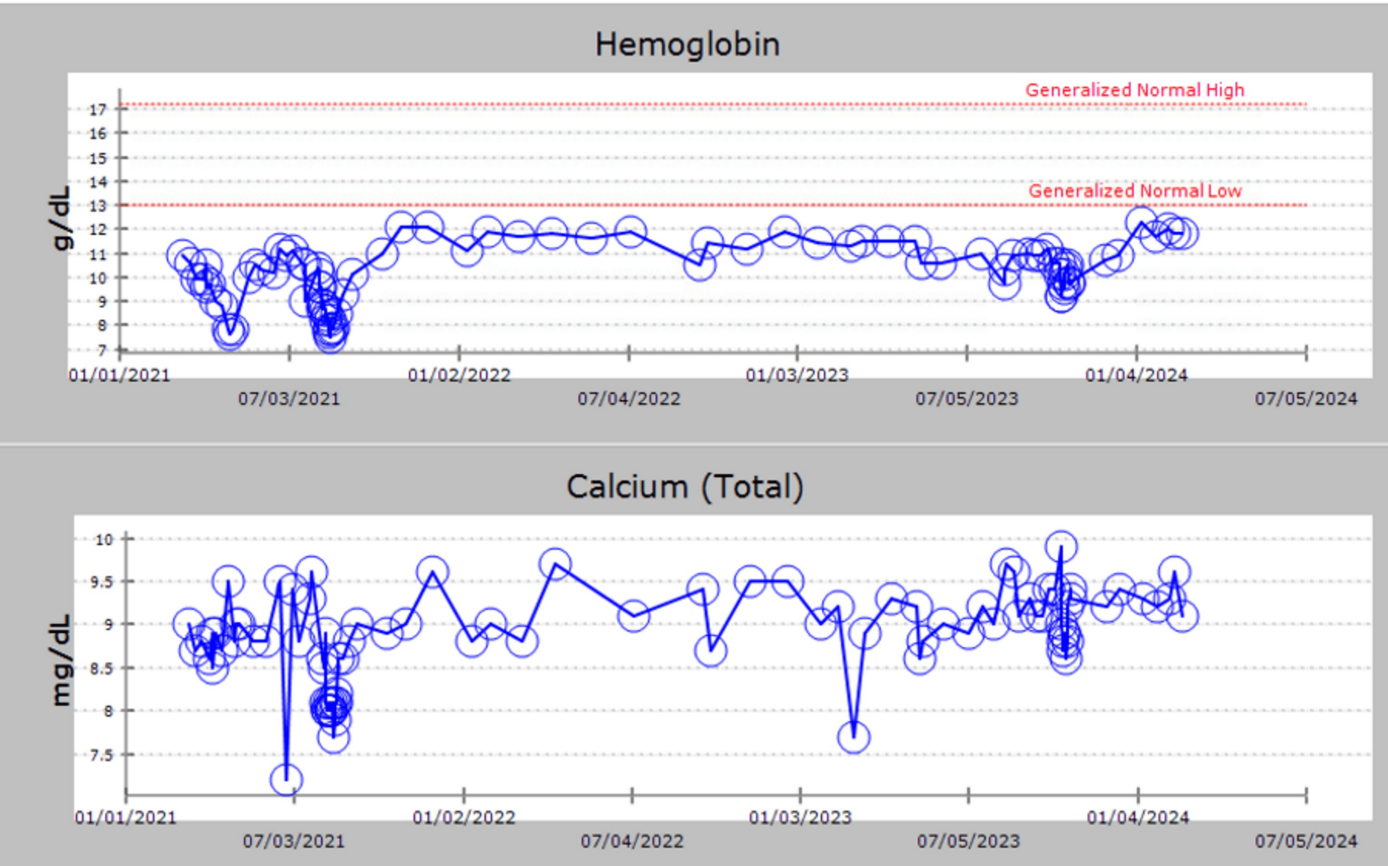
Hemoglobin and calcium levels were monitored throughout this period, indicating a comprehensive assessment of hematologic and metabolic parameters.

**Figure 2:**
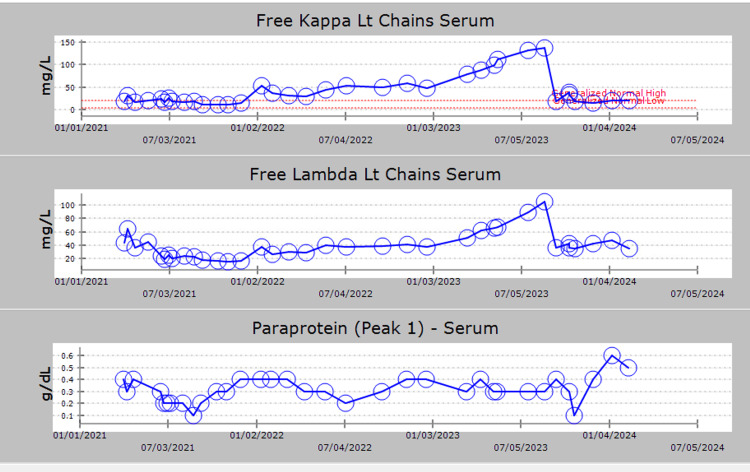
Free kappa light Chains and free lambda light Chains exhibited peaks between 07/05/2023 and 01/04/2024. Paraprotein levels in the serum showed an increase around 01/04/2024, indicating potential disease progression.

**Figure 3:**
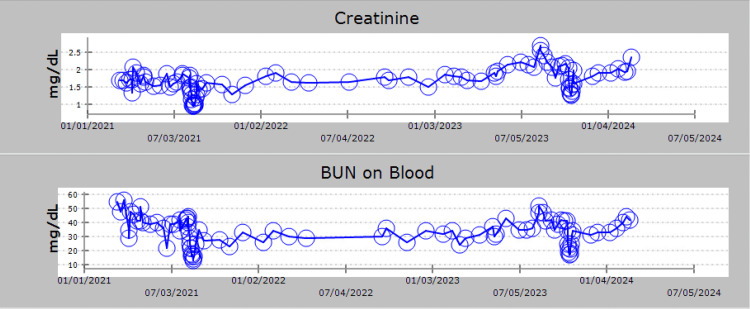
Renal function was monitored over the course of hospital admissions, with evidence of renal functional decline observed starting on 10/19/2023.

However, in April of 2023, the patient's renal function started to decline, and kappa light chains increased, suggesting a possible progression of the patient’s multiple myeloma (Figures 1-3). Orders were placed for bone marrow biopsy and whole-body PET CT scan to assess this possibility. The bone marrow biopsy done in May of 2023 revealed a hypocellular marrow showing trilineage hematopoiesis with full maturation and minimal residual disease, implying a lack of myeloma progression in the patient (Table 1). However, the PET CT scan revealed widespread nodular mass-like areas with intense lung fluorodeoxyglucose (FDG) uptake. This indicates airspace opacities consistent with consolidation likely due to an infectious or inflammatory process. As a result of the abnormal PET CT finding, repeat imaging was recommended 8-12 weeks later, and the patient was instructed to follow up with a pulmonologist who instructed the patient to follow up in six months.

**Table 1:** Flow cytometry analysis of the patient's bone marrow on 5/16/2023 was negative. The analysis demonstrated full maturation, with no residual cancer cells detected in the sample. All events recorded were individual cells (singlets), denoting a lack of cell aggregates or debris. Specifically, the Minimal Residual Disease (MRD) level was reported as 0.0000%, signifying the absence of detectable residual cancer cells. This outcome suggests a favorable response to treatment and a diminished risk of disease recurrence.

Population	Percent	Events
Ungated events	100	765133
Total singlet events analyzed	81.00	619778
% viability	97.19	9719
MRD % of singlets	0.0000	0
Total plasma cells	0.42	2214
Plasma cell % Kappa	56.27	1082
Plasma cell % lambda	43.73	841

**Figure 4:**
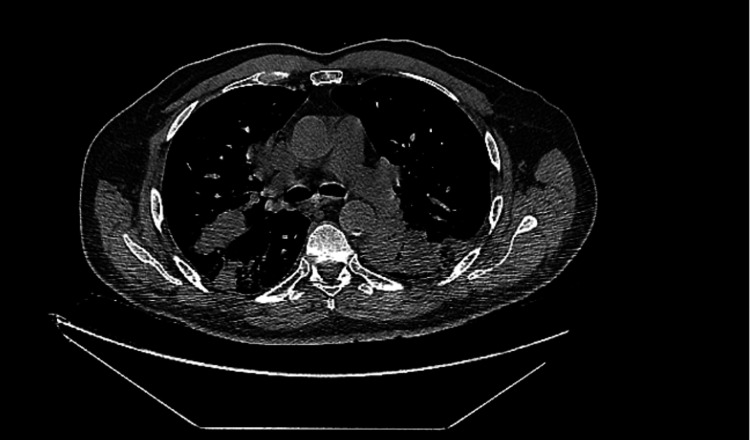
Bilateral lung consolidations were suggestive of infectious, inflammatory, or neoplastic etiologies.

In August of 2023, the patient presented to the emergency department complaining of sudden onset abdominal pain, chills, and fatigue. A CT scan performed in the emergency department showed bilateral lung consolidations suggestive of an infectious, inflammatory, or neoplastic process that has progressed since the initial scan in May of 2023 (Figure 4). Additionally, an abdominopelvic CT scan showed a distended bladder with mild hydronephrosis. Although hospital admission was recommended for further evaluation of the patient, the patient opted for outpatient management instead. The emergency department physician prescribed 100 mg of Doxycycline Hyclate to be taken twice daily for seven days, and the patient was discharged.

In the outpatient setting, the patient’s renal function continued to decline despite intravenous hydration. He also experienced significant weight loss, a decline in appetite, and a continuous increase in his kappa light chains (Figure 2). The medical team determined that the patient was refractory to Lenalidomide and was experiencing disease progression. The patient’s therapy was changed to Daratumumab, Pomalidomide, and Dexamethasone in August 2023.

Subsequent laboratory testing indicated a good response to the change in therapy, as light chains and renal function had significantly improved. However, a repeat CT scan of the chest in September of 2023 showed minimal improvement in the bilateral lung consolidations when compared to scans from August. Concerns were raised for possible non-infectious etiologies such as an organizing pneumonia or lymphoma.

In October of 2023, the patient presented to his Daratumumab infusion visit with a cough and sore throat but was afebrile. He was also neutropenic (0.7K/uL), tachycardic (110 BPM), and hypertensive (150/69 mmHg). The scheduled Daratumumab was not administered, and he was started on Cefepime 2 grams intravenously. A respiratory viral panel was negative (including CMV, EBV, HHV6, adenovirus, bacterial cultures, and fungal stains), and the patient received an additional chest CT scan, which showed interval mass-like consolidative opacities throughout both lungs. The patient was transferred to the emergency room and was treated empirically with Cefepime 2g IV every 12 hours, Azithromycin 500 mg daily, Fluconazole 400 mg daily, and Acyclovir 800 mg twice daily. Azithromycin was stopped, and the patient was started on Linezolid 600 mg IV every 12 hours for seven days to treat methicillin-resistant Staphylococcus aureus (MRSA) bronchitis pneumonia due to the patient testing positive in sputum culture. Fluconazole was escalated to Micafungin 50 mg IV daily. The CD4 count was 319 (41%), the CD8 count was 347 (45%), and the CD4/CD8 ratio was .98, indicated an immunocompromised state.

Atypical bacterial and fungal serologies were drawn, and blood titers for the cryptococcal antigen came back positive at a ratio of 1:256. A subsequent lumbar puncture assessing for meningeal involvement of the infection was negative, and the patient was placed back on Fluconazole 800 mg daily. Cefepime was discontinued, and the patient was placed on antibacterial prophylaxis with Levofloxacin 500 mg 1 tab daily since he was neutropenic.

It was recommended the patient continue oral Fluconazole 400 mg daily for six months and was discharged home. The Daratumumab was delayed for an additional four weeks to allow the patient to recover from the cryptococcal infection.

A repeat CT chest was done in January of 2024, which showed mild improvement in the lung consolidations. The patient resumed Daratumumab, Pomalidomide, and Dexamethasone to continue treating the myeloma. The patient remained stable and responded well to the treatment for the fungal infection. The patient continued follow-up with the infectious disease team, where the Quantitative Cryptococcus antigen remained undetectable, and the Cryptococcus antigen titer was 1:512. The patient has been asymptomatic, and his multiple myeloma is under control.

## Discussion

It is well established that patients with multiple myeloma are at increased risk for infectious diseases due to immunodeficiency. In the earlier stages of the disease, particularly within the first four to nine years after diagnosis, bacterial and viral infections are most common. Invasive fungal infections typically occur much later in the disease course, likely due to accumulated immunodeficiency from significant periods of aberrant immunoglobulin production and immunosuppressive MM therapy [1].

Other cases of Cryptococcus infection have been reported in patients with multiple myeloma. In a literature review of a similar case study, it was shown that in those with MM and a cryptococcal infection, 36% of patients had undergone prior transplantation for MM treatment, 43% had undergone multiple lines of treatment, and 75% were treated with pomalidomide and concomitant corticosteroids. This case study reviews the particular use of pomalidomide, a TNF-alpha inhibitor, which is known to impair CD4 T-cell activation. The use of pomalidomide causes decreased activation of CD4 T-cells, leading to increased susceptibility towards cryptococcal infection due to the altered cell-mediated immune response [1]. Pomalidomide, in combination with corticosteroids, may further increase the risk of invasive fungal infection in patients with multiple myeloma due to cumulative immunodeficiency.

Another case study involving disseminated cryptococcosis in a patient with MM treated with daratumumab, lenalidomide, and dexamethasone demonstrates that there are specific challenges in managing opportunistic infections in immunocompromised patients undergoing immunosuppressive therapies [5]. Despite continuous advancements in therapies for MM, novel treatments such as daratumumab, which targets CD38 in MM cells, may lead to immunosuppression [8]. The patient’s unfortunate outcome highlights the complexity of balancing disease control and infection risks in these patients. Similarly, the patient we present in this case was started on daratumumab due to being refractory to lenalidomide after the appearance of consolidations on PET-CT. However, following the initiation of therapy, the patient developed new-onset symptoms of weight loss, cough, and sore throat. Increased suspicion of cryptococcus fungal infections is necessary to optimize patient outcomes, especially when using immunomodulatory agents like daratumumab that have been shown to further compromise immune function [2,7]. Fortunately, our patient did not present with cryptococcal meningitis. This is noteworthy, as reported instances of disseminated cryptococcal infection have led to fatal outcomes [1,4-6].

Treatment of invasive cryptococcal infection involves induction therapy, consolidative therapy, and maintenance therapy. The induction stage of therapy involves the use of intravenous liposomal amphotericin B and flucytosine or fluconazole for approximately four to six weeks, depending on the meningeal involvement of the infection. The maintenance therapy involves fluconazole for at least one year to reduce the risk of disease relapse [9]. Although the patient in this case report was not treated with liposomal amphotericin B, they were treated with long-term fluconazole (400-800 mg) for six months.

## Conclusions

The case presented demonstrates that patients with MM are significantly susceptible to opportunistic infections due to aberrant production of ineffective immunoglobulins and immunosuppressive interventions. While cryptococcal infection is exceedingly rare in patients with MM, there is a need for heightened vigilance in clinical practice for these patients, especially with concurrent use of immunosuppressives. These findings align with existing literature, emphasizing the need for prompt diagnosis and rapid treatment of opportunistic infections, which account for significant mortality in MM patients. Physicians should maintain vigilance by assessing for infection in MM patients. These patients are often immunocompromised due to the disease itself and immunosuppressive therapies. Enhancing our understanding of the complexities of complications in MM is critical to reducing the high infection-related mortality in this population.
